# The impact and effectiveness of pneumococcal vaccination in Scotland for those aged 65 and over during winter 2003/2004

**DOI:** 10.1186/1471-2334-8-53

**Published:** 2008-04-23

**Authors:** John D Mooney, Amanda Weir, Jim McMenamin, Lewis D Ritchie, Tatania V Macfarlane, Colin R Simpson, Syed Ahmed, Chris Robertson, Stuart C Clarke

**Affiliations:** 1 Health Protection Scotland, Clifton House, Clifton Lane, Glasgow, UK; 2 Dorset and Somerset Health Protection Unit, Victoria House, Ferndown, Dorset, BH22 9JR, UK; 3 Department of Statistics and Modelling Science, University of Strathclyde, Glasgow, UK; 4 Department of General Practice, University of Aberdeen, Aberdeen, UK; 5 Department of Public Health, Greater Glasgow & Clyde NHS Board, Glasgow, UK; 6 Scottish Meningococcus and Pneumococcus Reference Laboratory, Glasgow, UK; 7 Molecular Microbiology Group, University of Southampton, Southampton, UK

## Abstract

**Background:**

For winter 2003/2004 in Scotland, it was recommended that all those aged 65 and over be eligible to receive 23-valent polysaccharide pneumococcal vaccine (23vPPV), which has been shown to be effective in reducing the risk of invasive pneumococcal disease (IPD). We assessed the success of the vaccination programme by examining the age specific incidence rates of IPD compared to four previous winter seasons and estimating vaccination effectiveness.

**Methods:**

Winter season incidence rates of IPD for vaccine targeted (65 years and over) and non-targeted (0–4, 5–34, 35–49, 50–64) age bands were examined for the Scottish population in a retrospective cohort design for winter 2003/2004. Details of all IPD cases were obtained from the central reference laboratory and population vaccine uptake information was estimated from a GP sentinel practice network. Based on the preceding four winter seasons, standardised incidence ratios (SIR) for invasive pneumococcal disease were determined by age-band and sex during winter 2003/2004. Vaccination effectiveness (VE) was estimated using both screening and indirect cohort methods. Numbers needed to vaccinate were derived from VE results using equivalent annual incidence estimates for winter 2003/2004.

**Results:**

Overall vaccination effectiveness using the screening method (adjusted for age and sex) in those aged 65 and over was 61.7% (95%CI: 45.1, 73.2) which corresponded to a number needed to vaccinate of 5206 (95%CI: 4388, 7122) per IPD case prevented. Estimated effectiveness for the same age group using the indirect cohort method was not significant at 51% (95%CI: -278, 94). Reductions in the winter season incidence rate of IPD were highly significant for all those aged 75+: males SIR = 58.8 (95%CI: 41.6, 80.8); females SIR = 70.0 (95%CI: 55.1, 87.8). In the 65–74 years age-group, the reduction for females was significant: SIR = 60.3 (95%CI: 39.3, 88.4), but not for males: SIR = 74.8 (95%CI: 50.8, 106.3). There was no significant protective effect on mortality.

**Conclusion:**

The introduction of 23vPPV for those aged 65 and over in Scotland during winter 2003/2004, was accompanied with a reduction of around one third in the incidence of IPD in this age group. Vaccination effectiveness estimates were comparable with those from other developed countries.

## Background

*Streptococcus pneumoniae *is one of the leading causes of bacteraemia and meningitis in the United Kingdom and in public health terms remains one of the most important bacterial pathogens worldwide [1]. While the incidence of invasive pneumococcal disease (IPD) is highest in very young children, the elderly and persons with underlying medical conditions are also at increased risk and mortality is highest in these groups [2,3]. In developed countries in particular, the combination of an aging population and rising levels of pneumococcal resistance to commonly used antibiotics have focused attention on preventative vaccination [4].

The current pneumococcal polysaccharide vaccine (23vPPV), which consists of 23 serotype antigens corresponding to over 90% of all invasive disease isolates, has been available since the early 1980's [5]. Its efficacy was first established against pneumococcal pneumonia in randomized controlled trials conducted amongst novice gold-miners in South Africa [6]. The balance of the evidence from a subsequent wealth of prospective and retrospective studies in immuno-competent older adults [7] together with cost effectiveness evaluations in the US [8] and Europe [9] tends to support the targeting of older age groups for vaccination as a worthwhile and cost saving intervention. The most recent Cochrane review also concluded that 23vPPV was effective against IPD although the evidence was not sufficient against pneumonia [10].

In winter 2003/2004, 23vPPV was recommended for all those aged 65 and over in Scotland and promoted in parallel with an influenza vaccination programme for the same age group [11]. This approach was at variance with the phased three year introduction programme for ten year age-bands (beginning with those aged 85 and over) that was adopted in England and Wales and completed in 2005/2006. The experience seen in Scotland may therefore serve as an early indication of the UK wide impact of the programme. Previous to the age targeted campaign, 23vPPV vaccine had only been recommended for all persons over the age of two years who were at increased risk of IPD due to any of the following underlying medical conditions: asplenia or splenic dysfunction; chronic renal disease or nephrotic syndrome; immunosuppression resulting from disease or treatment; chronic heart disease; chronic lung disease; chronic liver disease including cirrhosis and diabetes mellitus [12]. The two principal outcome measures by which the impact of the vaccination campaign was assessed in this evaluation were firstly the extent to which there was a reduction in the expected winter incidence of invasive disease in the target age-groups, since this was the major rationale behind the policy and secondly, the estimated vaccination effectiveness for those age 65 and over. During the time period of this evaluation, 23vPPV was the only population level age-targeted intervention against pneumococcal disease in Scotland since the 7-valent pneumococcal conjugate vaccine (Prevenar) was not introduced into the childhood vaccination programme until September 2006 [13].

## Methods

### Study design, population and time period

**Figure 1:**
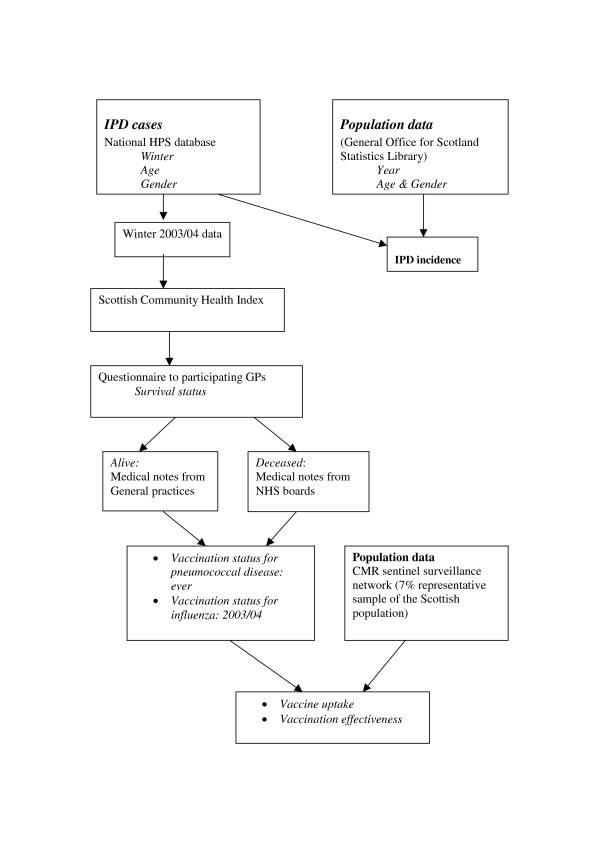
Study design and data sources.

The impact of the pneumococcal vaccination campaign in winter 2003/2004 was evaluated using a retrospective cohort design which looked at vaccination effectiveness and the age-specific incidence of IPD. The principle outcome measures were observed changes in the 2003/2004 winter season incidence rates of IPD in the vaccine targeted population of those aged 65 and over (divided into males and females aged 65–74 and 75 and over). For comparison, the incidence rates of invasive disease in younger age bands (0–4, 5–34, 35–49 and 50–64) were also examined for the same winter season. Figure 1 gives an overview of the study design and data sources.

### IPD incidence

Reports of all pneumococcal laboratory isolates from every diagnostic laboratory in Scotland are collated at Health Protection Scotland (HPS). In an ongoing collaboration with the Scottish Meningococcus and Pneumococcus Reference Laboratory (SMPRL), surveillance for invasive pneumococcal disease (IPD) involves obtaining laboratory confirmation, serotype identification and antibiotic resistance profiling for all blood and CSF isolates. There were no changes in culture procedures or in the criteria used by diagnostic laboratories to submit isolates over the period of the study. Laboratory reports of pneumococcal infection for the winter seasons (weeks 40 of preceding year to week 20 of the following year) were extracted from the national HPS database for 1999/2000; 2000/2001; 2001/2002; 2002/2003; 2003/2004. Total IPD isolates (i.e.: blood and CSF) for these time periods were used to derive winter season incidence rates by age-band (0–4, 5–34, 35–49, 50–64, 65–74 and 75+) and sex. For ease of comparison with other published studies, crude annualised incidence rates were estimated by assigning a two-thirds weighting to the winter season total (according to the mean proportion of annual cases which occur during weeks 40 to 20).

Population totals were obtained from the General Register Office for Scotland Statistics Library [14], the midyear estimates preceding each winter season being used as denominators. To verify any continuing trends, incidence rates for winter 2004/2005 were also determined. A Poisson regression model, using the log (population) as an offset variable was used to predict the expected number of cases for winter season 2003/2004, from which were derived standardised incidence ratios (SIR). The Poisson model used data on the incidence rates of the four preceding winter seasons and included the following terms: winter season (continuous variable), age group, sex and the interaction term of sex and age. The 95% confidence interval (CI) for the SIR was calculated using the error factor and a Chi-square test was used to compare numbers of observed and expected cases.

### Vaccine uptake

Estimates of 23vPPV vaccine uptake in winter 2003/2004 across the whole population were assessed through a sentinel surveillance network called the continuous morbidity record (CMR), which covers a seven percent representative sample of the Scottish population [15]. In contrast, influenza vaccine uptake data is not dependent on sample population projections, since it is based on item of service returns collated by the information and statistics division of NHS Health Services Scotland [16]. For every patient with laboratory confirmed IPD in winter 2003/2004, the Scottish community health index (CHI) [17] was used to obtain the details of their general medical practitioner/family doctor (GP). A postal questionnaire was then sent to confirm the patient's disease outcome, age, vaccination status with respect to both pneumococcal disease (ever vaccinated) and influenza (for the 2003/2004 season) and the presence of any underlying medical conditions deemed to present an increased risk of IPD [12]. For deceased patients whose records were no longer held within primary care, permission to access medical notes was sought in writing from the respective NHS boards (via Caldicott guardians). IPD cases were regarded as true vaccine failures if the date of vaccination was at least 14 days prior to the sample date, or the GP reply confirmed they had been vaccinated before the onset of their illness. For ethical purposes the patient follow up work was not classified as research, but as an evaluation of an ongoing vaccination programme. Chi-square tests for proportions were used to compare differences in 23vPPV uptake between sexes and different risk groups.

### Estimation of vaccination effectiveness

Estimates of vaccination effectiveness (VE) using the screening method described by Farrington [18] were derived by age-band (all 65+; 65–74 and all 75+) and sex using CMR uptake data projected across the whole population. Stratified estimates were obtained after separating patients classified as very high risk (VHR) which incorporated asplenics and the severely immuno-compromised (haematological malignancies and non-haematological malignancies who are currently receiving cancer therapy). VE results were also expressed as numbers needed to vaccinate per IPD case prevented. In addition, the indirect cohort method [19], which compares the proportions of pneumococcal infections caused by 23vPPV serotypes between vaccinated and unvaccinated IPD cases, was also used to obtain an estimate of VE. The method assumes vaccinated persons to be at the same risk of non-vaccine serotype infections as unvaccinated persons and cross-reactivity was assumed for serotypes closely related to vaccine components (e.g. 6A, 15C). Since the cohort effectively covers the whole Scottish population and survival outcomes were available from follow up, the relative risk of dying from pneumococcal disease could be calculated according to vaccination status for the target age-groups. An 'IPD related fatality' (i.e. most likely to have been a result of the pneumococcal disease episode) was defined as a case where death had been reported as the outcome on receipt of the sample by SMPRL, or where death was subsequently established to have occurred within 14 days of sample date. Mortality within 14 days of first positive blood sample corresponds to the time period adopted by the International Pneumococcal Study Group in relation to death from pneumococcal bacteraemia [20].

## Results

### IPD incidence in winter season 2003/2004

There were a total of 442 cases of IPD in Scotland during the study period, 170 of which occurred in those aged 65 and over. These correspond to equivalent annual incidence rates of 11.7 per 100,000 overall and 31.1 per 100,000 for the 65+ age group. For the model used to calculate standardised incidence rates there was no reason to reject the hypothesis that the Poisson regression and the included terms fitted the data (goodness-of-fit test P = 0.679). In winter 2003/2004 the observed incidence of IPD cases was significantly less than expected (on the basis of trends over the preceding four winter seasons) for the 65+ age group as a whole (34% reduction: SIR = 66.4; 95%CI: 56.8, 77.2) (Table 1). For specific age-bands within the 65+ population, there was a significant reduction for the 75+ age group of both sexes (male SIR= 58.8; 95%CI: 41.6, 80.8 and female SIR = 70.0; 95%CI: 55.1, 87.8) and for females in the 65–74 age group (SIR = 60.3; 95%CI: 39.3, 88.4). Among vaccine targeted age bands, the only reduction that did not achieve statistical significance was for that males aged 65–74 (SIR = 74.8; 95% CI = 50.8, 106.3).

**Table 1:** IPD Standardised Incidence Ratios (SIR) for winter 2003/2004 by age-band and sex

**Age group ** **(years)**	**Observed ** **cases**	**Expected** **cases**	**SIR**	**95% CI**	**Chi- ** **square** **test P-** **value**	**Equivalent** **annual** **incidence** ** rates/** **100,000**
**65+**						

**Males**	69	106	65.2	50.7 – 82.6	<0.001**	30.85
**Females**	101	150	67.1	54.7 – 81.6	<0.001**	31.33

**65+ All**	170	256	66.4	56.8 – 77.2	<0.001**	31.13

**Males**						
0–4	36	30	118.4	82.9 – 164.1	0.310	40.04
5–34	37	25	150.2	105.7 – 207.2	0.013*	5.79
35–49	37	39	95.0	66.8 – 131.1	0.757	9.98
50–64	42	44	95.8	69.0 – 129.56	0.780	14.03
65–74	31	41	74.8	50.8 – 106.3	0.105	22.74
75+	38	65	58.8	41.6 – 80.8	<0.001**	43.49

**Females**						
0–4	27	18	152.2	100.2 – 221.8	0.028	31.41
5–34	32	16	204.8	140.0 – 289.4	<0.001*	5.05
35–49	26	20	129.4	84.4 – 189.8	0.188	6.62
50–64	35	42	83.9	58.4 – 116.8	0.298	11.16
65–74	26	43	60.3	39.3 – 88.4	0.009**	15.74
75+	75	107	70.0	55.1 – 87.8	0.002**	47.72

* Significant increase from expected incidence rate produced by mode.

** Significant decrease from expected incidence rate produced by model

### Vaccine uptake

Vaccine uptake estimates for the general population as obtained at the beginning of December 2003 from CMR practices were 68.1% for males and 65.5% for females (Table 2). This figure had altered little by the end of vaccination uptake monitoring when the overall figure based on returns from general practice was 65% [21]. Of the 145 65+ IPD cases for whom vaccination status was known, 63(43.4%) had received pneumococcal vaccine (34 out of 66 (51.5%) male cases and 29 out of 79 (36.7%) female cases). The proportion of vaccine recipients among 65+ IPD cases was not significantly different between those with or without respiratory disease (45% vs. 42.5% respectively, p = 0.760); those with or without cardiovascular disease (50% vs. 37.9%, p = 0.182) or those with or without 'more than one risk factor' (46.1% vs. 42.3%, p = 0.823).

**Table 2:** Percentage vaccine uptake by age-band and sex in CMR sample and IPD cases

	**CMR**		**IPD**	
	
**Age**	**Male** % vaccine uptake (total number in group)	**Female** % vaccine uptake (total number in group)	**Male** % vaccine uptake (total number in group^#^)	**Female** % vaccine uptake (total number in group^#^)
Age group (years)				
0 – 4	0.3% (9,181)	0.2% (8,697)	7.7% (26)	5.3% (19)
5 – 34	0.8% (89,636)	0.9% (86,147)	8.0% (25)	5.6% (18)
35 – 49	2.7% (42,428)	3.0% (41,461)	4.5% (22)	3.7% (27)
50 – 64	9.9% (32,724)	10.5% (32,673)	9.4% (32)	2.9% (34)
65 – 74	66.7% (13.887)	65.3% (15,866)	53.8% (26)	42.3% (26)
75+	70.5% (8,744)	65.7% (14,907)	50.0% (40)	34.0% (53)

**All age 65+ years**	68.1% (22,631)	65.5% (30,773)	51.5% (66)	36.7% (79)

**Very high risk**	-	-		
No			22.4% (161)	18.3% (164)
Yes			60.0% (10)	23.1% (13)

# Excluding unknown vaccination status

**Figure 2:**
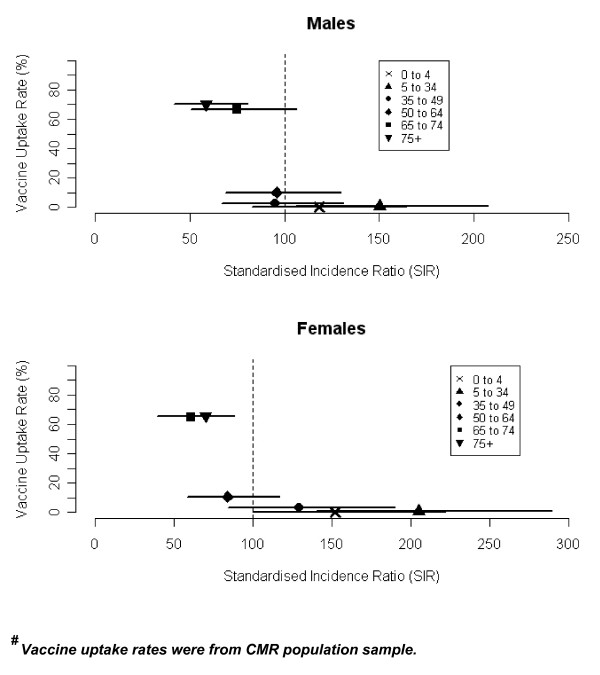
Standardised incidence ratios (SIR) for invasive pneumococcal disease in winter 2003/04 with 95%CI limits and correlation with vaccine uptake rates^# ^by age-band and sex.

Vaccine uptake rates in the CMR sample population were inversely associated with the SIRs for both men and women (Figure 2). The significant reduction in the SIR was only seen among vaccine targeted age groups with a correspondingly high vaccine uptake. Out of a total of 448 isolates, 417 were successfully matched to patient details through their community health index numbers and after exclusion of non-retrievable and mismatched records, 396 (88.4%) were available for follow up. Of those matched records, 348 were completed for vaccination status and for the presence of underlying risk factors including 145 (87.9%) of the 65+ cases. Table 3 shows the baseline statistics of completed records linked to patients with regard to sex, age and proportion of VHR. Overall, 75 (21.6%) IPD cases had received pneumococcal vaccination, with date of vaccination known for 71 (94.7%). Of these, 43 (60.6%) had received vaccine in 2003 or 2004. Out of a total of 60 23vPPV vaccine recipients in patients aged 65 or over, 52 (86.7%) had also received influenza vaccine for the 2003/2004 winter season. Among IPD patients, the percentage who had received vaccine in all age groups was generally higher for males compared to females (Table 2), although the difference was not statistically significant (p = 0.180). A slightly higher vaccine uptake for males aged 65+ was also evident in the CMR sample population. For the 65+ population as a whole, there was a steady increase in the uptake of influenza vaccine over the course of the study period, rising from 64.9% in winter 2001/2002 to 68.9% in 2002/2003 and 72.5% in 2003/2004 [16].

**Table 3:** Baseline characteristics of IPD cases linked to patient records

**Description**	**IPD Cases linked to patient records**	**Completed Follow-up (%)**	**Vaccination status known (%)**	With >1 Risk factor (%) *
Number of subjects	396	368	348	61
Male (%)	198 (50.0)	181 (49.2)	171 (49.1)	28 (45.9)
Aged 65 + years (%)	165 (41.7)	156 (42.4)	145 (41.7)	43 (70.5)
% VHR^§^	-	29 (7.9)	23 (6.6)	-

* With >1 risk factor: more than one known risk factor for pneumococcal disease as set out in non-age dependent criteria to be eligible for polysaccharide vaccine [12].

^§ ^VHR denotes IPD cases in the very high risk category – defined as asplenics and the severely immuno-compromised (haematological malignancies and non-haematological malignancies who are currently receiving cancer therapy).

### Vaccination effectiveness

The overall result obtained for crude vaccination effectiveness (VE) for the 65+ age group was 64.2% (95% CI: 49.6, 74.5) and after adjustment for age and sex, the revised estimate was 61.7% (95% CI: 45.1, 73.2). The adjusted figure corresponds to a number needed to vaccinate (NNV) to prevent one IPD case of 5206 (95%CI: 4388, 7122). Table 4 shows VE results with 95% confidence limits for those aged 65+, 65–74 and 75+ by sex after the exclusion of the VHR category. Sex differences in effectiveness are apparent for the 65–74 age band at 64.6% for females (95%CI: 21.1, 84.1) versus 41.0% (95%CI: -31.7, 73.6) for males, although these differences as well as effectiveness in males were not statistically significant. Estimated VE for the VHR subgroup who were aged 65 and over was also not significant at 37% (95%CI: -80, 70). The very low numbers of non-vaccine component strains in IPD patients (five out of the six cases being in 65+ patients, three of whom were vaccinated) means that the VE estimate by the indirect cohort method was associated with a high range of uncertainty and did not reach statistical significance (VE (65+) = 51% (95% CI:-278, 94). Since the majority of IPD patients who had received pneumococcal vaccine also received the current seasonal influenza vaccine, patient numbers were insufficient to obtain a reliable estimate of vaccination effectiveness for pneumococcal vaccine alone.

**Table 4:** Vaccination effectiveness in those age 65 years and over (excluding very high risk) with equivalent number needed to vaccinate (NNV) estimates

**Group**	**Number of vaccinated cases**	**Total number of cases**	**% Vaccination Effectiveness (95% CI)**	**Equivalent Number Needed to Vaccinate **^#^**(NNV) with 95% CI**
Age 65+ years				
All				
Crude	57	135	64.2 (49.6, 74.5)	-
Adjust			61.7 (45.1, 73.2)	5206 (4388, 7122)

(Age/Sex)	30	59	53.5 (22.5, 72.1)	6059 (4499, 14407)
Male	27	76	71.1 (53.7, 81.9)	9835 (3987, 5944)
Female				
Age 65–74 years				
All	23	49	54.4 (20.1, 74.0)	9724 (7148, 26317)
Male	13	24	41.0 (-31.7, 73.6)	*(10726) *§
Female	10	25	64.6 (21.1, 84.1)	9835 (7554, 30110)

Age 75+ years				
All	34	86	68.8 (52.0, 79.8)	3145 (2712, 4162)
Male	17	35	60.5 (23.3, 79.6)	3801 (2895, 9891)
Female	17	51	73.9 (53.3, 85.4)	2836 (2454, 3932)

* Vaccination effectiveness as determined by the screening method (see Methods)

# NNV: number needed to vaccinate to prevent one case (calculated as 1/attributable risk; based on equivalent annual incidence of IPD in 2003/2004.

§Hypothetical value for NNV given non-significant test result

### Serotype analysis and vaccine failures

Serotype confirmation was obtained for 388 (98%) of the 396 IPD isolates received in winter 2003/2004. Of the 109 IPD cases aged 65+ for whom serotype was confirmed, 46 out of 48 (95.8%) of those vaccinated had 23vPPV strains as did 56 out of the 57 (98.2%) who had not been vaccinated. Of the three non-vaccine serotypes, serotypes 16 and 31 were found in vaccinated cases and serotype 34 in a non-vaccinated case. For the whole dataset of IPD cases, 71 could be described as 'true vaccine failures', nine of whom were classified as being very high risk ('VHR', see methods). A further 20 patients were known to have had more than one risk factor and overall 26 of the 71 (36.6%) were aged over 80 yrs.

### Vaccination and IPD survival

There were 70 IPD related fatalities overall, 50 of which occurred in those aged 65 and over and in which vaccination status was obtained for 44. Omitting the six cases without vaccination details, the crude case fatality rate in the 65 plus age group was 27.0% for vaccinated patients and 32.9% for those who had not been vaccinated. Based on estimated population uptake data, this translated into a NNV to prevent one IPD related fatality of 14810 (Table 5). Since the reduced risk of dying as a result of pneumococcal vaccination was not statistically significant, the NNV estimate can only be seen as hypothetical. Crude relative risk reductions by age-band and sex for those aged 65 and over (based on pneumococcal vaccination status alone), ranged from 0.71 to 0.96 though none were statistically significant. There was also no difference by sex or age-band (65–74 versus 75+).

**Table 5:** Pneumococcal vaccination status and relative risk (RR) of mortality in patients aged 65 years and over

**Group**	**Total no. in group**	**% Died**	**RR Mortality (95% CI) Crude**	**RR (95% CI) adjusted for age group and gender**	**NNV^§ ^to prevent 1 death**
**All age 65+ years**					
No vaccine	82	32.9	1.00	1.00	14,810
Vaccine	63	28.6	0.86 (0.47, 1.56)	0.89 (0.49, 1.63)	

**Age group (years)**					
*65–74*					
No vaccine	27	22.2	1.00	**-**	39,172
Vaccine	25	16.0	0.71 (0.20, 2.51)		
*75+*					
No vaccine	55	38.2	1.00	**-**	8,122
Vaccine	38	36.8	0.96 (0.49, 1.89)		

**Gender**					
*Males*					
No vaccine	31	32.3	1.00	**-**	
Vaccine	34	29.4	0.90 (0.38, 2.17)		
*Females*					
No vaccine	51	33.3	1.00	**-**	
Vaccine	29	27.6	0.81 (0.35, 1.89)		

^§^**NNV: **Number needed to vaccinate to prevent 1 death. Note: since the reduction in mortality risk was not significant, these are hypothetical estimates included only for comparison purposes.

## Discussion

The introduction of 23-valent pneumococcal polysaccharide vaccine for those aged 65 and over in Scotland in winter 2003/2004 was accompanied with a significantly reduced burden of invasive disease for this age group and an estimated VE comparable with results seen elsewhere [22-24]. Although other studies have demonstrated the effectiveness of this intervention in reducing the incidence of IPD, high levels of chronic disease morbidity in Scotland [25] that are not simply confined to qualifying medical conditions, meant that automatic assumptions about the likely success of such a campaign were not possible at the outset. Preliminary incidence data for winter 2004/2005, showing a continued reduced incidence of IPD in the 65+ population (equivalent annual incidence rate of 30.0/100,000 compared with 31.1 for winter 2003/2004), is further testimony to the apparent success of the programme. A marginal though inconclusive reduction in mortality risk was also suggested for IPD cases aged over 65 who had been vaccinated, although this was not statistically significant for the one winter season analysed here. The NNV to prevent one case of IPD in this evaluation (5206) is considerably lower than the recent Cochrane review estimate of 20,000 although this was calculated for a 55 years and over population where incidence would be much lower and is also based on a 50% vaccine effectiveness [10]. Since the NNV estimates given here also used equivalent annual incidence rates after the implementation of the vaccine programme, they can be considered to be conservative.

While invasive pneumococcal disease accounts for only a small component of the morbidity from the pneumococcus, it is at the more severe end of the clinical spectrum and it is the outcome for which the evidence is strongest in showing a protective effect from 23vPPV [26]. There is also a lack of ambiguity about a laboratory isolate, which is not the case for 'all cause pneumonia' or 'all cause mortality' which have also been used as indicator outcomes for assessing VE against pneumococcal infection [27]. Additionally since the major rationale for the pneumococcal vaccination campaign was aimed at reducing invasive disease and its associated mortality and burden on health services, this was believed to be the most legitimate means by which to evaluate the programme's success or failure.

Observational studies can be said to assess the effects of health care interventions without influencing the care that is provided or the patients who receive it [28]. When used in the assessment of vaccination programmes therefore they have high external validity and broad generalisability. In addition a number of randomised trials which have sought to assess the effectiveness of 23vPPV have been insufficiently powered to detect real benefits and have therefore proven inconclusive, particularly with respect to rare outcomes such as IPD [29]. Non-randomised studies such as the current evaluation are limited by the extent to which there may be dissimilarities between vaccinated and non-vaccinated persons, in both their likelihood of receiving vaccination and in their subsequent care and follow up. Our findings that IPD patients with respiratory disease, cardiovascular disease or more than one underlying chronic health condition were no more likely to have received vaccine that those who did not, provides some reassurance in this regard. Further reassurance can be derived from the presumption that if vaccine recipients are more likely to be in poorer health, then the reported benefits in this study would be an underestimate rather than an overestimate of VE.

There are benefits as well as drawbacks to the evaluation of a pneumococcal vaccine campaign based on just a single post-implementation winter season. While the effects remain subject to between season variability according to temperature, airborne pollutants and circulating respiratory viruses [30], the short time frame also minimises differences in effect that may arise from changes in circulating serotypes and variability in the duration of antibody response, which has been acknowledged as a problem in elderly vaccine recipients [31]. With respect to circulating respiratory viruses, the retrospective inclusion of all four previous winter seasons benefited from known low levels of influenza (at or below seasonal baseline based on GP 'flu-spotter' consultations) in each of those seasons and during the 2003/2004 season of 23vPPV implementation [32]. Marginal increases in influenza vaccination uptake over the study period are therefore unlikely to have influenced the disease burden of IPD. The fact that almost all vaccinated IPD patients also received influenza vaccine however means that we cannot exclude the possibility that there was some additive benefit of both vaccines as has clearly been demonstrated by other authors [33,34]. The one season post-implementation study design may also yield an over-optimistic evaluation of VE that is not likely to be sustainable, due to both declining antibody levels and the increased likelihood with time of encountering non-vaccine component serotypes [35]. Additionally, the modest scale of the Scottish population makes it feasible to centrally collate all cases of invasive pneumococcal disease allowing for completeness of reporting and comparability between different years and winter seasons. Retrospective ascertainment of vaccination status is of course less dependable than prospective clarification, but the use of GP records is more reliable than self-reporting methods [36] as is the electronic recording of uptake rates in the sample CMR population.

In spite of our results being restricted to one winter season, it has been possible to derive VE values in those aged 65 and over that compare well with other studies that have also looked at IPD prevention [23,37]. A high vaccine uptake in the first season of implementation also means that any observed effect on the burden of disease is not likely to be blurred by an annually increasing uptake rate, as has been postulated for the US [38]. While substantial overlap between the confidence intervals makes it difficult to interpret the variation in VE by sex and age-band, the finding of the highest effectiveness being in the 75+ age category contrasts with the diminishing effectiveness with age generally seen in other studies. For the Scottish population however an element of survivor bias may be relevant, since the majority of those surviving to 75+ years of age are from higher socio-economic groups [39]. The higher effectiveness in females aged between 65 and 74 in spite of lower vaccine uptake, may reflect higher levels of underlying chronic illness in males of this age group (although this is not evident from available risk factor data in this study, Table 3). More severe underlying illness has also been linked with an increasing the likelihood of opting for vaccination [40]. A slightly higher uptake of the pneumococcal plus flu vaccine in older males, which is a combination known to have additive benefits [33], may also have contributed to the high effectiveness in this sub-group although, as noted previously, seasonal influenza activity was low throughout the study period. The current estimate of VE by the indirect cohort method is characterised by a high range of uncertainty although the actual value of 51% for those aged 65+ is not incompatible with a protective effect. The multiple infecting serotypes of the pneumococcal pathogen together with the high valency of 23vPPV mean that reliable estimates using this method, such as that derived by Butler in the US [41], commonly require large numbers of cases (specifically around 2400 gathered over a period of 14 years in that particular study). Single winter season estimates such as our own or that determined in a recent Australian study [22] inevitably have low numbers of rare non-vaccine serotypes which introduces a large margin of error.

The parallel targeting of pneumococcal vaccine with seasonal influenza has been shown to be an effective means of achieving high uptake in pneumococcal programmes for older adults [42] and the estimated overall uptake levels in the 65+ for the season under study (at 65%) also reflect the suitability of primary care as a means of delivery. The recent Australian evaluation [22] reported reductions in IPD incidence (36%) of similar magnitude to that seen in Scotland and VE estimates in the same range as for the current study (71%: 95%CI: 54, 82). Similarly overall VE estimates obtained from a case control study in Catalonia [23] were also in the same range (65%: 95%CI: 35, 81). These comparisons add a measure of confidence to the generalisability of our results to other developed countries.

The inherent complexity of the pneumococcus and it's sophistication in evading the host immune response, requires that a high degree of vigilance at both the epidemiological and microbiological levels continue to accompany the ongoing implementation of this vaccine programme for older adults. A key issue in its medium to long term success will be the duration of protective effect which several studies have shown to decline rapidly in elderly subjects and in a manner that is not uniform across all serotypes [27,35]. The extent to which the elderly population may have gained additional protection from the introduction of 7-valent conjugate vaccine (PCV-7) to the childhood schedule from September 2006 [13] mediated through a herd immunity effect [43], and as has shown promise in the US [44], awaits further investigation. Protection against invasive disease is of course only one aspect of tackling pneumococcal morbidity and the extent to which polysaccharide vaccine, either alone or in combination with the new conjugate vaccines, might be able to reduce pneumococcal pneumonia is the next key question in tackling the societal burden of this pathogen. The evidence with regard to pneumonia is certainly less clear cut and may need to await the development of more specific and efficient diagnostic tests [45].

## Conclusion

The introduction of pneumococcal polysaccharide vaccine for those aged 65 and over in Scotland during winter 2003/2004 was associated with a reduction of around one third in the incidence of invasive pneumococcal disease in this age group. Given the epidemiological diversity of the pneumococcus by population and by region [4], policymakers ought to be encouraged that the VE estimates obtained are comparable to those seen in other developed countries. Additionally, as the first large scale demonstration of effectiveness in a UK population, the results should strengthen the evidence base for health care practitioners involved in distributing vaccine in England and Wales, now that the phased roll-out to all over 65s is complete. Joint influenza and pneumococcal immunization in the primary healthcare setting is both effective and widely acceptable as evidenced by high uptake rates, and should continue to be a mainstay of disease prevention for this age group in years to come. Whether the reduced incidence of invasive disease will persist for those aged 65 and over will only be apparent when data for future years/winter seasons are analysed. Of course any sustained change in serotype distribution towards non-vaccine component serotypes may yet have significant implications for the current vaccination programme and its longer term effectiveness.

## Competing interests

JDM received financial assistance from Sanofi Pasteur MSD Ltd to present this work at the 12th International Congress on Infectious Diseases in Lisbon (June 2006). All other authors declare that they have no competing interests.

## Authors' contributions

JDM wrote the manuscript and subsequent revisions, conducted reviews of the literature and carried out the initial preliminary analysis. AW formulated the regression model, carried out the incidence rate analysis and partly wrote the methods and results sections. JM oversaw the study design and interpretation. LDR co-ordinated the contribution of Aberdeen University Department of General Practice and advised on the structure of the paper. TVM checked the statistical analysis and advised on interpretation of the results. CRS analysed vaccine uptake rates and advised on data collection and interpretation from primary care. CR critically reviewed the methods and results sections and suggested changes before submission. SA provided advice on vaccination policy context and contributed to the design stage. SCC oversaw the laboratory analysis of patient isolates and provided expertise on the molecular epidemiology of the pneumoccocus. All authors read and approved the final manuscript.

## Pre-publication history

The pre-publication history for this paper can be accessed here:


